# “They are talking from the Encyclopedia Britannica brain”: diabetes patients’ perceptions of barriers to communicating with physicians

**DOI:** 10.1186/s12913-020-5063-4

**Published:** 2020-03-13

**Authors:** Howard S. Gordon, Lisa K. Sharp, Antoinette Schoenthaler

**Affiliations:** 1 Jesse Brown Veterans Affairs Medical Center and VA Center of Innovation for Complex Chronic Healthcare, Chicago, IL USA; 2 Section of Academic Internal Medicine and Geriatrics, Department of Medicine, University of Illinois at Chicago, MC 275, 1747 W. Roosevelt Ave, Chicago, IL 60608 USA; 3 Institute for Health Research and Policy, University of Illinois at Chicago, MC 275, 1747 W. Roosevelt Ave, Chicago, IL 60608 USA; 4 Department of Pharmacy Systems, Outcomes & Policy, College of Pharmacy, University of Illinois at Chicago, Chicago, IL USA; 5 Center for Healthful Behavior Change, Department of Population Health, NYU School of Medicine, New York, NY USA

**Keywords:** Patient participation, Type 2 diabetes mellitus, Patient-physician relations, Veterans, Qualitative research

## Abstract

**Background:**

Understanding patients’ beliefs about their role communicating in medical visits is an important pre-requisite to encourage patients’ use of active participatory communication, and these beliefs may be particularly relevant for patients with diabetes.

**Methods:**

Focus groups were conducted to examine patients with diabetes view of their role communicating in medical encounters. Patients had type 2 diabetes, A1C ≥ 8% (64 mmol/mol), and were from an inner-city VA hospital. Guiding questions for the focus groups were based on theoretical models of patient-physician communication. Focus group transcripts were analyzed with the constant comparative method.

**Results:**

Four focus groups were conducted with a total of 20 male Veterans. Participants mean age was 61 years, 65% self-identified as black or African-American, 80% completed high school or higher education, and mean A1C was 10.3% (89 mmol/mol). Eight themes were identified as to why patients might have difficulty communicating with physicians. These themes were grouped into three overarching categories explaining reasons why patients might avoid participatory communication and included patients’ view about their condition; about physician’s communication behaviors; and about external influences on patient-physician communication. For example, patients described how use of the EHR may deter patients’ use of active participatory communication.

**Conclusions:**

These results are important for understanding how patients’ use of active participatory communication is influenced by their beliefs and expectations, physicians’ behaviors, and structural factors. The results may be useful for educational efforts to increase patient, physician, and healthcare systems awareness of problems that patients perceive when communicating with physicians.

## Background

Patients’ beliefs about their role in medical interactions with physicians are fundamental to their subsequent communication behaviors [1]. Beliefs about whether it is the patients’ role to adopt specific communication behaviors (e.g., ask the physician questions) and beliefs or expectations that their communication will be taken into account, help determine whether patients assume an active communication style during medical encounters [1, 2]. Focusing on the patient role is important because communication in medical visits is a mutual endeavor [3]. When patients use active communication – for example: ask questions; express opinions; or make requests – most physicians will respond according to norms of communication with an answer that is topically related [2]. Thus, active patient communication is powerful because it can influence physicians’ communication. Patients beliefs about their role communicating with their physicians within medical visits is especially salient for patients with chronic conditions like type 2 diabetes [4]. Research among patients with chronic disease has shown that patients who take an active role in communication are more satisfied, receive more information, and exhibit better health outcomes [5–7].

**Fig. 1:**
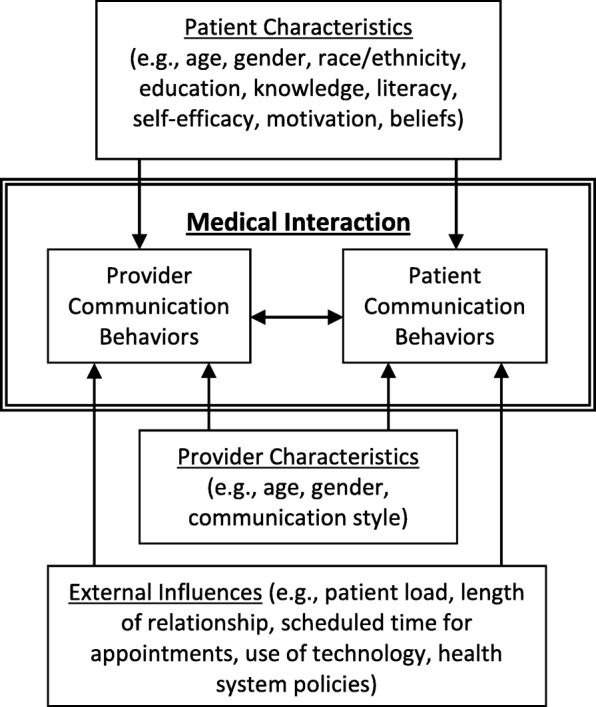
Patient, provider, and external influences on the patient role communicating in medical interactions

Adopting an active patient role and overcoming communication barriers in medical visits may be more challenging for patients with chronic disease, low health literacy, and for vulnerable populations [8–11]. According to several models, patients’ communication in medical encounters is influenced by the patient’s individual attributes, the physician’s characteristics and style, and the external context of the encounter (see Fig. 1) [12–14]. Patients must have confidence to speak up and must believe it is acceptable to initiate active communication [15]. Individual attributes such as motivation and self-efficacy are needed for patients to produce active communication [16]. Yet, competing patient, physician, and external contextual factors, such as patient information overload or discomfort, can suppress talking about sensitive topics and preclude use of active communication behaviors. Common communication challenges, such as not knowing what questions to ask or fears the physician will react defensively, may limit the effectiveness of communication in medical visits [12, 14, 16]. Further, when patients’ beliefs or physician behaviors discourage an active patient role, it can be challenging to convey information, particularly if patients believe their questions will waste the physician’s time or if physicians do not provide the conversational space for patients to speak up. Thus, reliance on physicians to actively inform and to prompt patients to engage in the conversation is often not sufficient [17].

Understanding the barriers to patients’ active communication in medical visits is an important step in efforts to develop interventions to improve patients’ skills and confidence when communicating with health care providers. Therefore, the authors undertook a qualitative study using focus group interviews to examine beliefs that influence patients active participation in a racially diverse population with type 2 diabetes mellitus (T2DM) and hemoglobin A1c ≥ 8% (64 mmol/mol).

## Methods

### Participants

The electronic health record (EHR) at an inner-city Veterans Affairs (VA) hospital was used to identify patients with T2DM, a hemoglobin A1c (A1C) > 8% (64 mmol/mol), and at least 2 visits in the primary care internal medicine clinic in the prior year. An A1C > 8% (64 mmol/mol) was chosen to capture patients whose T2DM was not controlled and for whom most providers would recommend intensification or increased adherence to medication, diet, and exercise regimens. The study was described to 48 patients and 20 provided written informed consent and attended a focus group. There was no significant difference in mean age (*P* = 0.30) or mean A1C (*P* = 0.76) for patients who attended a focus group compared with those not attending. Participants were provided parking validation and $40 for their time. Participant demographics were self-reported. This study was approved by the Jesse Brown VA Medical Center Institutional Review Board (#751255).

### Focus group interviews

The focus group guiding questions and prompts were based on conceptual models of patient-physician communication and were developed to encourage discussion about patients’ preferences, needs, and goals for communicating with their physician and the internal and external factors influencing their communication [2, 12, 13]. The moderator began each group with a welcoming message and the ground rules for discussion (e.g., maintaining confidentiality, avoiding interruptions). A short video entitled “Questions are the Answer,” from the United States Agency for Health Research and Quality, was shown as an ice-breaker and to introduce the topic of “asking questions.” Group discussions followed a standard format using an interview guide of semi-structured questions. The introductory guiding questions focused on diabetes: 1. the shared experience of having diabetes, 2. discussions about their diabetes diagnosis, and 3. what it is like to have diabetes. The main guiding questions focused on physician-patient communication about diabetes: 4. How do you feel about talking to your doctor? 5. Sometimes patients may not understand what the doctor is saying. How do you decide whether you ask your doctor to explain things? 6. What do you think would make it easier for you to talk to your doctor? 7. We want to help patients and doctors talk to each other, what do you think about that? Questions were followed with prompts for clarification and additional explication. Groups were conducted in a private conference room, lasted one to 2 hours, and were facilitated by one of the authors who is experienced in qualitative research. An assistant kept notes and managed the audio-recorders.

### Analysis

The analysis of the interviews began after the first interview was transcribed. Using the constant comparison method fundamental to grounded theory analysis, themes were generated independently by each coder. The themes represented patient perspectives on the salience of factors associated with patients’ active participatory communication. The two coders then met to discuss each transcript on a line-by-line basis and to refine and agree upon the themes. Themes were grouped into categories based on our model (Fig. 1) from several communication frameworks [12–14]. Coherence, credibility, and strength of those interpretations was achieved with multidisciplinary (physician, psychologist) triangulation among the coders. Member checks verified the trustworthiness of patient education materials developed using the themes generated in this study.

## Results

Participants (*N* = 20) from the four focus groups had poorly controlled T2DM with mean A1C of 10.3% (89 mmol/mol). Mean age was 61 years (SD 6.7), all were male, most participants self-identified as black or African-American, and more than half of participants had at least a high school education (Table 1).

**Table 1:** Patient Characteristics

Mean Age in years	61 ± 6.7 (Range 48–78)
Race	
African American	65%
White	25%
American Indian or Asian	10%
Education	
College Degree	25%
Some College	40%
High School or Equivalent	15%
Less than High School	20%
Mean A1C (%)	10.3 ± 2.5 (range 8.1–17.9)

### Qualitative analysis

Analysis of focus group transcripts yielded eight themes that patients identified as difficulties in communicating with physicians. Each of these themes is defined in Table 2. The themes are described below with quotes illustrating patients’ perceived barriers to active participatory communication. The themes are grouped according to communication frameworks (Fig. 1) into one of three categories of barriers that influence physician-patient communication. The first two categories comprise two groups of themes classified as influences internal to the medical interaction: one being how patients’ feelings and beliefs about themselves or their condition influence communication, and the other how patients’ perceptions of the physician influence patients’ communication in the medical interaction. The third category includes themes related to external influences on the communication in the medical interaction.

**Table 2:** Categories (in italics) and Themes developed from focus group transcripts

*Patients’ self-constructed beliefs about their condition*	
Minimizing Severity	Minimizing the importance of T2DM and self-management
Emotional/Motivational	Shame or self-blame about motivational challenges self-managing T2DM
*Perceptions of Physicians Behaviors*	
Testing the Relationship	Participants described difficulty understanding the physician and used their assessment to determine whether to remain active.
Pressure	Participants indicated that a physician used a lecturing/controlling style to direct behavior change.
Power – Distance	An imbalance of power in the medical interaction and a resulting relational distance (separation) between patient and physician.
*Perceptions about external influences on the medical encounter*	
Lack of Continuity	Problems with not knowing the physician and not having a physician who knows you.
Electronic Health Record (EHR)	Participants felt the computer diverted the physician’s attention and participants made assumptions about information in the EHR.
Time	Participants believed that physicians were busy and that time constraints limited communication

### Participants’ self-constructed beliefs explain their communication

Themes related to participants’ feelings and beliefs reflected self-blame about meeting the challenges of self-management or minimization of the severity of their diabetes. Participants who minimized the severity of their condition justified their communication behaviors by creating a positive reframing of the bad news that their diabetes was not controlled. These are exemplified by their own modified definitions of acceptable blood glucose control, for example:

*“[My sugar] is a little bit high. Never went over 400, so I consider that good.” (P3, FG4)*

*“I'm doing what I'm supposed to do with my diabetes and I'm not doing bad with my diabetes. My diabetes could be a lot worse.” (P4, FG3)*

In addition, participants expressed shame and embarrassment that their diabetes was not controlled, identifying a lack of motivation to adhere to the recommended diabetes treatment regimen. Patients felt they were “messing up”, for example:

*“Sometimes I’m ashamed about how I’ve been carrying on. I know I eat the wrong things. I know I don’t exercise and I know my sugar’s way too high.” (P2, FG3)*

These feelings of shame left some participants unable to talk freely with the physician about difficulties they were having controlling their behaviors as illustrated in these examples:

*“The thing is, I don't feel comfortable [asking questions] because, really it's my fault.” (P4, FG4)*

*“I know that part of the reason is because, I eat, and I eat something with sugar before I go to bed. And that's why it's so high. I'm not going to talk to my doctor about trouble with my insulin because I know it's my own fault.” (P1, FG1)*

Patients preferred to remain silent rather than discussing challenges with diet and medication.

### Participants’ role was influenced by perceived or expected physician behaviors

Participants’ statements referring to the physicians’ behaviors included testing the relationship, pressure, and power-distance. Participants were Veterans and frequently chose descriptive terms that related to their military experience (e.g., “battling”) when describing communication with physicians. Participants described how physicians’ use of medical jargon or too much information was a barrier to active participation in the encounter.

*“I'm battling an encyclopedia … They are talking from the Encyclopedia Britannica brain.” (P1, FG3)*

Participants indicated that they evaluated the physician’s style of communication to determine their continued use of active participation. When they perceived the physician was using language they did not understand, particularly in response to a question, they would tend to withdraw. In this way, participants tested the relationship with the physician, for example:

*“…the way they respond to the question that I asked. If I don’t think or understand the response they gave me, then I don’t go any further with it. If I understand, then it goes to a different color.” (P1, FG3)*

*“Well, I didn’t understand what they were saying and I didn’t want any more bad news so I left it at that.” (P3, FG3)*

Statements that were coded as ‘pressure’ indicated that participants objected to the physician’s authoritarian tone, identifying physicians’ communication style as negatively paternalistic and illuminating the active distancing that occurs due to feeling pressured. Pressure was a common theme exemplified by participants’ reports that physicians used warnings or threats to discuss a medical condition. The perceived conflict or tension in the medical encounter interfered with communication and was a reason for not speaking up for many participants.

*“They use scare tactics and try to intimidate you and like I keep thinking we were all boot camp mentalitied [sic] into not being afraid of death.” (P5, FG3)*

The third theme “power-distance” was related to the patients’ perception that the physician was controlling the visit. Patients perceived a power struggle in the physician-patient relationship where the physician holds the information and determines how the visit unfolds. Statements described how distancing and disengagement were used when participants exerted their own form of control by deciding not to engage. In this way patients shielded themselves from perceived power struggles, for example:

*“[Doctor] shoots at me and I just, bulletproof, I just take it. There's no negotiating with [Dr.]” (P4, FG3)*

Similarly, others described a strategy of asserting their own power to withhold information or provide misinformation thereby creating more distance.

*“I say what I think they want to hear … I’ll tell them a lie; I just say ‘Yeah, everything's fine’.” (P1, FG3)*

### Participants’ perceptions about external influences on the medical encounter

Several themes about barriers to communication were related to the clinical context of care. These barriers were external influences on the medical interaction and included three themes: lack of continuity of care, use of the EHR, and patients’ perception that there was not enough time available for the medical encounter.

Participants’ statements about lack of continuity of care were marked by frustration about difficulty establishing a personal relationship with their physician. The statements reflected a desire to be known by their physician. Discontinuities of care – especially when physicians turnover – were an impediment to developing a therapeutic relationship. Without time to develop trust, patients may feel less comfortable with the new doctor.

*“You get comfortable with a certain doctor and then they change you and they give you a new doctor. And then you have to start all over again.” (P5, FG2)*

Statements about the computer in the exam room or about use of the EHR reflected perceptions about how technology was a barrier to communication by interfering with getting “*some eye contact*” and making participants feel “*ignored.*” Participants also commented on how technology might discourage their active participatory communication and thereby perpetuate passivity.

*“[Dr.] already knows … my body … because the test they have taken [are in the computer] so I just really answer [the doctor’s] questions.” (P2, FG3)*

Participants believed that they did not need to tell the physician about themselves because the information was “in the computer” or were frustrated when they had to repeat something to a new physician because the “previous doctor doesn’t put [it] in there.”

Time was another theme that was commonly raised. Time constraints were seen by patients as a pre-determining factor that negatively impacted their experience with the physician as illustrated by the following statement:

*“The other thing is, you get the sense of being taken care of but at the same time being rushed because they have to see the next guy because when you came in to see them, you saw 25 other guys in the chairs waiting. It’s an unspoken situation, but I’m going to take care of you but it’s only going to take 32 seconds because I have the next 32 seconds to take care of the next guy.” (P5, FG3)*

The perceived brevity of the encounter was seen by one patient as the physician’s disinterest and was used as a rationale to justify patients’ lack of communication.

*“Well, if people are not going to take time with you, then that turns you off and you’re not going to ask questions if they don’t want to have time.” (P1, FG4)*

Participants also described feeling pressured to not prolong the encounter with their questions or concerns, thus perpetuating their own withdrawal, desiring only to answer the physician’s questions to avoid bothering them, for example:

*“It’s that unmentioned sensation that you get. I need to ask questions, but I don’t want to ask questions because it may be too complicated and too long.” (P5, FG3)*

Finally, the theme of time-related barriers also included a sense of fatalism; for example, this participant expressed the belief that the physician might use his time better with another patient and that it was futile to speak up:

*“Just accepting that no matter if I ask or not, it's going to be what it's going to be. So why worry [the doctor], [Dr.] can see someone else.” (P3, focus group #4)*

## Discussion

Our analysis identified eight themes that patients described as challenges to their use of active participatory communication in the medical interaction. In our synthesis of these results the eight themes were grouped into three categories according to our communication model (Fig. 1): (1) patients’ beliefs about their condition; (2) patients’ perceptions or expectancies about physicians’ communication behaviors; and (3) patients’ perceptions about external contextual factors that influence patient-physician communication. Understanding these barriers is important because patients who are less engaged and use less active participatory communication may gain less understanding of their T2DM, may be less likely to adhere to treatment recommendations and may have worse outcomes [18, 19]. These results may be useful in the development of communication skills training interventions to encourage patients to more actively communicate with their physician and to train physicians to improve skills in partnering with patients during medical encounters.

Our study reports a novel and interesting finding that participants seemed to overestimate the completeness of content in the electronic health record (EHR). This assumption that information about them was or could be obtained from the EHR was connected to a belief that an active patient role was less important because the physician already “knew” them. These results suggest that patients’ overestimates of the accessibility and completeness of computerized content precludes patient participation and perpetuates patient passivity. To prevent this unintended consequence of EHR use, physicians could integrate the EHR into the visit so that it augments rather than impedes communication [20, 21].

Our study is also novel in highlighting participants perceived power differential with their providers. The descriptive military references provided by Veterans with diabetes in our study gives their unique perspective on communicating with providers. Participants choice of words such as battling, bulletproof, shoots at me, and boot camp mentality describe a confrontational style of encounter where patients object to provider behavior and perceive they must oppose or retreat rather than partner with provider. Patients reported how they adjust their role by testing the relationship and if they object to the subsequent provider behavior patients react by shielding themselves or asserting their own power, which interfered with effective communication in the encounter.

Our results may be especially important when considering that active patient participation can be more challenging for racial/ethnic minorities. In particular, African-American patients may be less likely to speak up than white patients and in turn get less information from their physicians [8–10]. This may be related to a physician-patient power differential. Physicians have more knowledge and information than patients and may use a controlling and authoritarian style that inhibits patients active participation [17]. African-American patients (and other minorities) often have an added communication challenge related to lower sociocultural power because they are more likely to be seen in racially discordant medical interactions [22]. As our results indicate, when patients perceive the physician is using a controlling or authoritarian style, they may decide to assert their own control by withholding information or providing misinformation [17, 23]. Patients also may be less likely to speak up when they are facing challenges self-managing their condition and in turn feel shame or blame themselves. Those feelings may be reinforced when providers do not acknowledge the challenges and offer support with self-management. Lack of trust in the provider as well as the longer amount of time needed to develop a trusting relationship may also explain the racial differences in African-American patients’ active communication style [24, 25]. Moreover, discontinuities of care could be more frustrating to African-Americans, particularly in teaching hospitals, low resource areas, or other settings where physician turnover is high.

Our other findings are consistent with prior research on physician-patient communication. For example, other studies have reported that patients deny or minimize the severity of their T2DM and that physicians’ use a lecturing or paternalistic style when evaluating self-management behaviors [26, 27]. Similarly, studies have found that patients value physician continuity for coordination of care and for developing trusting relationships [28, 29]. Our finding that patients withdraw in the face of medical jargon instead of speaking up to ask questions for clarification is consistent with a reported relationship between poor health literacy and worse communication behaviors [30]. Studies have also identified inadequate time as a barrier to patient-physician communication [31].

Several limitations should be noted. First, our results are from an inner-city sample of male US Veterans and may not generalize to women, patients with other conditions, or different settings of care. Second, it is possible that participation in the study was biased by the financial incentive or wherein those who were dissatisfied with their care were more likely to participate. Third, with data from four focus groups it is possible we did not achieve complete saturation of themes. That is, it is possible that additional focus groups would have identified additional themes. Nonetheless, our study design and patient sample had features consistent with sufficient information power [32]. Features of information power in our study include that our participants were homogeneous (male Veterans with elevated A1C), our interviews contained rich quality of dialogue, our study was informed by established communication theory [12–14], and our aim was narrowly focused on patient perceptions about communication with providers.

## Conclusions

The detailed patient descriptions about decisions to communicate in medical visits is a strength of this study. Many studies use an alternative quantitative method such as the patient activation measure to assess patients’ communication with physicians [33]. Yet, surveys measuring patients’ ratings of communication with their physicians are poorly correlated with external observers’ ratings of patients actual behaviors, when those observers listen to a recording of the interaction and use the same rating scale [34, 35]. Thus, evaluation of patient activation using patient ratings alone will not provide a complete assessment of patients’ use of active participatory communication. Additional methods such as those used in this study provide important information about how patients decide when to use active communication behaviors.

These qualitative results provide insight into how we can help patients prepare to be more active and speak up in medical visits, how we can help health care professionals become more aware of and to avoid or overcome their behaviors that limit patients active communication, and how to work around and remove structural factors that may inhibit good communication. One example of a structural factor is our interesting and novel finding about the interaction of exam room EHR use and patient-physician communication which may lessen patients’ active participation. This result highlights how external structural factors may influence patient-physician communication and may be used to suggest how system re-design could support active patient communication, for example, by allowing screen sharing content from EHR. These results also provide data that could help train providers to minimize jargon and bridge power differentials [36] and encourage patients to actively participate.

Because our study focused on patients’ perceptions, our findings may be particularly suited to inform the design and development of pre-visit educational materials that dispel myths, improve self-efficacy to communicate, encourage patients to adopt an active patient role, and demonstrate with role models how to use active participatory communication behaviors to overcome barriers to communicating in medical visits. For example, our results indicate that patients may not speak up if they believe the physician is in a rush or that information is already in the EHR. Pre-visit educational interventions that inform patients, for example, that physicians want to know their concerns or that all the details are not in the EHR and interventions to provide communication training to health professionals and to reduce structural barriers, would be important prompts to encourage patients to take an active role.

Previously developed interventions to encourage patients’ active communication have shown improved processes and outcomes of care, including active communication, treatment adherence, functional status, and biomedical outcomes; however, the most effective interventions used trained personnel and as a result were expensive [5–7]. Perhaps because of the expense, such interventions are not widely implemented. Alternative methods are needed for the delivery of interventions that encourage an active patient role in medical visits. Video-based interventions provide a potential alternative method. Video-based education is acceptable to patients from a broad range of cultural backgrounds and can be particularly useful for patients with limited literacy [37]. Direct-to-consumer video-based television advertisements are used effectively in the United States by the pharmaceutical industry. These ads influence patient behavior and activate patients to make specific requests in medical visits. Interventions that use visit planning materials to prepare patients to speak up and to alert physicians to expect patients’ questions and concerns may be effective [38, 39]. More research is needed to understand the full potential of these pre-consultation visit planning interventions, which have the advantage of being significantly less expensive than interventions requiring coaching personnel, and may be more easily disseminated than coaching interventions. Furthermore, interventions that encourage active patient communication may in turn influence physician behaviors. Because communication is a two-way street – interventions that improve patients’ communication have the potential to improve physicians’ communication. For example, due to norms of communication, physicians will generally respond when patients ask questions or use other active communication behaviors. In summary, our results provide data that may support future research and quality improvement efforts in three areas: preparing patients to speak up; alerting physicians to their behaviors that may discourage patients communication; and identifying structural factors for interventions that reduce barriers to patients’ use of active participatory communication.

## Data Availability

Upon request, the corresponding author will consider requests for the final dataset. These requests for access will be reviewed by the Jesse Brown VA Research & Development committee and Associate Chief of Staff for Research and addressed within a reasonable timeframe. The dataset will include deidentified data relevant to the specific request.

## Abbreviations

A1C: Hemoglobin A1c
EHR: Electronic Health Record
FG: Focus Group
T2DM: Type 2 Diabetes Mellitus
VA: Veterans Affairs
